# Hepatocellular Senescence: Immunosurveillance and Future Senescence-Induced Therapy in Hepatocellular Carcinoma

**DOI:** 10.3389/fonc.2020.589908

**Published:** 2020-11-27

**Authors:** Peng Liu, Qinghe Tang, Miaomiao Chen, Wenjian Chen, Yanli Lu, Zhongmin Liu, Zhiying He

**Affiliations:** ^1^Institute for Regenerative Medicine, Shanghai East Hospital, School of Life Sciences and Technology, Tongji University, Shanghai, China; ^2^Shanghai Institute of Stem Cell Research and Clinical Translation, Shanghai, China; ^3^Department of Hepatobiliary and Pancreatic Surgery, Shanghai East Hospital, Tongji University, Shanghai, China

**Keywords:** cellular senescence, hepatocellular senescence, senescence-associated secretory phenotype, immunosurveillance, senescence-induced therapy, hepatocellular carcinoma

## Abstract

Hepatocellular carcinoma (HCC) is the third leading cause of cancer-related deaths worldwide. The lack of effective targeted drugs has become a challenge on treating HCC patients. Cellular senescence is closely linked to the occurrence, development, and therapy of tumor. Induction of cellular senescence and further activation of immune surveillance provides a new strategy to develop HCC targeted drugs, that is, senescence-induced therapy for HCC. Precancerous hepatocytes or HCC cells can be induced into senescent cells, subsequently producing senescence-associated secretory phenotype (SASP) factors. SASP factors recruit and activate various types of immune cells, including T cells, NK cells, macrophages, and their subtypes, which carry out the role of immune surveillance and elimination of senescent cells, ultimately preventing the occurrence of HCC or inhibiting the progression of HCC. Specific interventions in several checkpoints of senescence-mediated therapy will make positive contributions to suppress tumorigenesis and progression of HCC, for instance, by applying small molecular compounds to induce cellular senescence or selecting cytokines/chemokines to activate immunosurveillance, supplementing adoptive immunocytes to remove senescent cells, and screening chemical drugs to induce apoptosis of senescent cells or accelerate clearance of senescent cells. These interventional checkpoints become potential chemotherapeutic targets in senescence-induced therapy for HCC. In this review, we focus on the frontiers of senescence-induced therapy and discuss senescent characteristics of hepatocytes during hepatocarcinogenesis as well as the roles and mechanisms of senescent cell induction and clearance, and cellular senescence-related immunosurveillance during the formation and progression of HCC.

## Introduction

The incidence rate of liver cancer has been increasing along with population growth and aging in the past years. In accordance with GLOBOCAN statistics, there were about 841,080 new cases of liver cancer and 781,631 liver cancer-related deaths worldwide in 2018 (1). Hepatocellular carcinoma (HCC) is the fifth most common malignant tumor and the third leading cause of cancer-related death globally, accounting for 75–85% of primary liver cancer cases, and it has become a serious public health issue, especially in China, where the number of annual new HCC cases and HCC-related deaths accounts for about half of the whole world’s corresponding number (1–3). Among the most common cancers in China, the mortality of liver cancer ranks third in men and fourth in women respectively in 2017 (1, 3).

The occurrence and development of HCC is an irreversible process often accompanied by repeated hepatocellular injury, inflammation, necrosis, and regeneration. Up to 80% of HCC cases are associated with chronic liver injury, such as hepatitis virus infection, alcohol abuse, drug toxicity, and metabolic disorders, which gradually progress to liver fibrosis and cirrhosis, eventually resulting in HCC (1, 3–5).

Traditional treatments for HCC mainly include surgery (hepatectomy, liver transplantation, ablation, and intervention), chemotherapy, radiotherapy, and biotherapy. Among these approaches, surgery is most commonly used, but the high rate of metastasis and recurrence after operation has become the bottleneck to improve the prognosis of patients with HCC (6, 7). Currently, many new therapeutic strategies have been developed for the treatment of refractory or advanced HCC, such as molecular-targeted agents (e.g., Sorafenib and Lenvatinib), immunotherapy (immunomodulators, immune-checkpoint inhibitors, CAR-T, and HCC vaccines), and oncolytic virotherapy (6). However, the sensitivity, efficacy, and safety of these treatments are still clinically questionable, implying that the challenge of HCC therapy remains unsolved (8). For instance, intermediate- and advanced-stage HCC are not sensitive to drug therapy, which limits the clinical application of existing anti-HCC drugs (9). Although individualized treatment can be formulated according to different therapeutic methods, the five-year overall survival of HCC has not been effectively improved. Also, HCC is highly heterogeneous: analysis of a large number of HCC samples show that abnormal regulation of signaling pathways such as telomere maintenance, cell cycle control, Wnt/β-catenin signaling, chromatin modification, RTK-RAS-PI3K signaling, and oxidative stress in HCC cells (6), and the wide range of mutations or abnormal expression at the gene level. The heterogeneity in HCC greatly limits the effect of targeted drugs. Therefore, it is imperative to explore more appropriate solutions for HCC therapy.

In recent years, research on cellular senescence is gradually becoming a valuable and promising field since the correlation between age-related chronic liver diseases and senescence has been recognized (10, 11). In particular, cellular senescence is closely linked to the occurrence, development, and treatment of HCC (12). Despite much attention paid to the field, the contribution of cellular senescence to liver diseases and its precise mechanisms have not yet been clearly elaborated. Therefore, understanding the role of cellular senescence in the pathogenesis of HCC will establish theoretical and practical basis for clinical treatment of liver tumor. Of note, cellular senescence-related therapeutic strategy of HCC is gaining importance, which is achieved through the use of specific small molecular compound inducing cellular senescence, activating immunosurveillance, as well as eliminating or killing senescent precancerous hepatocytes and HCC cells in various ways. As a highly immunosuppressive tumor, the microenvironment makes it insensitive for HCC cells to respond to immune system monitoring (13, 14). Our recent study found that inducible cellular senescence showed highly effective on HCC suppression since senescent cells could remarkably activate immune surveillance and recruit multiple types of immune cells to infiltrate and remove atypical proliferative hepatocytes by the secretion of senescence-associated secretory phenotype factors (15). Taken together, this review will introduce the emerging senescence-relevant therapeutic methods of restricting HCC and discuss the future prospects and possible disadvantages of senescence-based therapy for HCC.

## Cellular Senescence and Its Antitumor Effect

### Characteristics of Cellular Senescence

In response to endogenous and exogenous stress, cells in various tissues may enter a state of cell cycle arrest. These cells are called senescent cells that cannot proliferate but remain metabolically active for an extended period of time (16). Abnormal telomere function in normal cells can result in replicative senescence. Harmful stimuli like oncogene overexpression, irreversible DNA damage, oxidative stress, and endoplasmic reticulum (ER) stress may individually or synergistically accelerate the senescence of normal cells (17). The transformation from normal cells to senescent cells runs through the whole life cycle of organisms and plays a key role in tissue homeostasis. Moreover, cellular senescence has been proved to be critically involved in specific physiological and pathological processes upon the conditions of stress signal stimulation, such as embryonic development, tissue repair, tumorigenesis, tumor suppression, and aging (18).

The phenotype of senescence is quite stable and persistent, showing no response to mitogenic stimulation and resistance to apoptosis, namely the ability of “senescence without death” to cause aging-related diseases (17). Senescent cells are characterized by changes in morphology and nuclear membrane, lysosome activity, and gene expressions, and present significant upregulation of cell cycle inhibitors such as p53/p21 and p16^INK4a^, activation of DNA damage response, remodeling of chromatin structures, deposition of senescence-associated β-galactosidase (SA-β-Gal), and induction of senescence-associated secretory phenotype (SASP). Full-fledged senescence phenotypes are usually manifested to be enlarged and flattened, and often multinucleated (19). Aravinthan et al. reported that senescent hepatocytes with high expression of p21 exhibited much larger nuclei than non-senescent hepatocytes (20).

Except for p21 and p16^INK4a^, SA-β-Gal and SASP are another two hallmarks of senescence, existing in almost all types of senescent cells. Determination of SA-β-Gal activity is the most commonly used method to detect cellular senescence (21). SASP is a generic term for all senescent cell-secreted components including a large amount of proinflammatory cytokines, chemokines, growth factors, and proteases, which contribute to senescence-related pathophysiological processes and evidently affect adjacent cells and their microenvironment in both autocrine and paracrine manners (17). Senescent cells are highly secretory and perform myriad SASP-mediated functions, the beneficial or detrimental effects of which depend on physiological context of the liver and other organs (22). These diverse activities of SASP composition include angiogenesis, activation and inhibition of cell proliferation, formation of chemoresistant niche in cancer chemotherapy, stimulation of epithelial-to-mesenchymal transition, induction of senescence, activation of inflammation and immunosurveillance, regulation of stem cell renewal and differentiation, as well as optimization of tissue repair (19, 23). Moreover, the regulation of SASP is affected by multiple pathways and molecular mechanisms. A significant proportion of SASP factors are positively regulated by p38 MAPK/MK2 signals, the DDR (DNA damage response) proteins ATM, NBS1, and CHK2 as well as the transcription factors NF-κB and C/EBP-β. In contrast, p53 negatively regulates or restrains the SASP (16). A summary of causes, characteristics, and effects of cellular senescence is shown in **Table 1** (19, 23).

**Table 1:** Summarization of cellular senescence.

Causes	Characteristics	Consequences
**·** Telomere shortening or dysfunction **·** Activation of oncogene (e.g., RAS) **·** Loss or inactivation of tumor suppressor (e.g., RB, PTEN, NF1, and VHL) **·** DNA damage **·** Epigenetic stress **·** Oxidative stress **·** ER stress **·** Proteotoxic stress **·** Nucleolar stress **·** Spindle stress **·** Low BubR1 **·** Others	**·** Permanent cell-cycle arrest **·** Apoptosis resistance **· DNA content:** 2N or 4N **· Increased:** Cell size; SA-β-gal activity; p16^INK4a^; p19^ARF^; ATM/R; p53/p21; p15^INK4b^; p27^KIP1^; ROS; p38 MAPK; DNA-SCARS; SAHF; γ-H2AX foci **· Decreased:** Telomere length; DNA synthesis (Ki67, EdU); Proliferation (CDK2/4/6) **· SASP factors:** Cytokines (IL-1α/1β, IL-6) Chemokines (IL-8, CCL2/MCP-1, CXCL1) Growth factors (bFGF, HGF, IGF, TGF-β, G-CSF) Proteases (MMP-1/3/13) **· Others:** Loss of Lamin B1 Enhanced NF-κB signaling	**In development:** **·** Embryonic development (e.g., morphogen gradients and changes in cellularity) **·** Placental angiogenesis **In adulthood:** ***Acute senescence*** **·** Tumor suppression **·** Tissue repair (e.g., wound healing) ***Chronic senescence*** **·** Tumorigenesis and progression **·** Tissue dysfunction **·** Aging-related degeneration or diseases (e.g., Alzheimer disease, Osteoarthritis, Type 2 diabetes, and Atherosclerosis)

ER, endoplasmic reticulum; SA-β-gal, senescence-associated β-galactosidase; ATM, ataxia telangiectasia mutated; ATR, ataxia telangiectasia and RAD3-related protein; ROS, reactive oxygen species; p38 MAPK, p38 mitogen-activated protein kinase; DNA-SCARS, DNA segments with chromatin alterations reinforcing senescence; SAHF, senescence-associated heterochromatin foci; SASP, senescence-associated secretory phenotype.

### The Protective Role of Cellular Senescence Against Tumorigenesis

As mentioned earlier, cellular senescence is closely related to tumorigenesis and refers to a relatively stable state where cells are irreversibly separated from cell cycle and lost the ability of proliferation due to persistent stressed injuries. Under the stimulation of carcinogenesis, usually the cells enter senescent state followed by cell cycle arrest and cell division suspension, and then tumorigenesis is inhibited (16). On the other hand, in early-phase of cancer, regulatory dysfunction occurs in senescence-related signaling pathways, making damaged cells fail to grow senescent normally and then cell cycle become uncontrolled (24). It can be seen that cellular senescence is a potential antitumor mechanism. Senescence-induced therapy for preventing oncogenesis means that artificially inducible senescent cells secrete proinflammatory SASP factors and further recruit a variety of immune cells such as T cells, NK cells, and macrophages and their subtypes to infiltrate around the lesion tissues and participate in the activation of immunosurveillance, quickly identifying and clearing senescent cells, and finally blocking tumorigenesis (13, 14, 25).

As a natural barrier for tumor inhibition, cellular senescence is regulated by p53/p21 and p16^INK4a^ signaling pathways (26). Upon the stimulation of mitogenic signals or cytokines, cyclins accumulate in early G1 phase and forms a complex with CDKs (cyclin-dependent kinases). This activated complex such as cyclin E-CDK2 or cyclin D-CDK4/6 initiates the phosphorylation of RB and then promotes the release of E2F transcription factors from RB, thereby driving the expressions of genes required for cells to enter the S-phase for mitosis (27, 28). CDK inhibitors p53/p21 and p16^INK4a^ can block their common downstream cyclins-CDKs-RB/E2F axis and antagonize G1-S progression by respectively targeting cyclin E-CDK2 and cyclin D-CDK4/6 complexes (19, 28).

Tumorigenesis is a long pathological process that gradually breaks the limitation of senescent mechanism. Normal cells accumulate a series of driving carcinogenic factors before becoming real cancer cells (26). Oncogene-induced senescence (OIS) is triggered by the activation of oncogene or inhibition of tumor suppressor gene (29). Due to the redundant regulation of cellular senescence pathways, cancer cells that break through the OIS limitations will still retain the response to senescent induction, which can also lead to the senescence of cancer cells after the treatment of senescent induction, namely therapy-induced senescence (TIS) (30). Many studies have achieved effective treatment of cancer by inducing cellular senescence. For example, CDK4 gene knockout could immediately induce the senescence of non-small cell lung cancer cells driven by K-Ras, thus resulting in tumor regression (31). CDK4/6 inhibitors specifically accelerate the induction of cellular senescence by inhibiting CDK4/6-induced phosphorylation of RB, subsequently activate immunosurveillance by SASP, and effectively clear senescent cancer cells including HCC cells (16, 28). Notably, as early as 2015, the specific CDK4/6 inhibitor Palbociclib has been approved by the U.S. Food and Drug Administration for clinical treatment of advanced breast cancer (32). The genotoxic drug Oxaliplatin could cause DNA damage and oxidative stress, which induced senescence in HCC cells as a form of senescence-induced therapy (33). Radiotherapy as the method of TIS can induce DNA damage, and further accelerate senescence or death of tumor cells including HCC cells under therapeutic dose (34).

Immune-checkpoint inhibitors, targeting programmed cell death-1 (PD-1), programmed cell death ligand-1 (PD-L1), or cytotoxic T-lymphocyte protein 4 (CTLA-4), can specifically block the corresponding immunosuppressive signals and exhibit potential efficacy on malignant tumor including advanced HCC (35, 36). Clinical trials have identified a manageable safety profile and durable antitumor responses of anti-PD-1 therapy in advanced HCC (6). Nonetheless, a relatively small number of responders can benefit from immune-checkpoint inhibitor monotherapy. Recently, an alternative antitumor schedule by the combination of immune-checkpoint inhibitors and CDK4/6 inhibitors has achieved clinical exploration and synergistic efficacy over the monotherapy. According to the approval numbers obtained from the ClinicalTrials.gov database, in addition to the use of single agent CDK4/6 inhibitors in anti-cancer such as anti-HCC treatment (Identifier: NCT01356628; NCT03109886; NCT02524119), several clinical trials combining CDK4/6 inhibitors (Palbociclib, Ribociclib, or Abemaciclib) with immune-checkpoint inhibitors for breast cancer (Identifier: NCT03294694; NCT02791334; NCT02779751), ovarian cancer (Identifier: NCT03294694), and non-small cell lung cancer (Identifier: NCT02779751) are ongoing or active (28).

Moreover, senescence-related immune surveillance plays an important role through SASP factors and infiltrating immunocytes such as CD4^+^ Th1 cells and M1 macrophages, which can promote the clearance of senescent precancerous cells and restrict tumor formation. Among them, CD4^+^ Th1 cells kill senescent cells by releasing IFNγ and TNFα, and M1 macrophages inhibit the proliferation of malignant cells and promote their apoptosis through TGFβ signal (37). It was also found that the combination of Palbociclib and Trametinib, a specific K-ras-targeted drug, could induce the senescence of lung cancer cells with K-ras mutation, and then SASP factors could activate natural killer (NK) cells, thus improving the treatment effect on lung cancer (38). In addition, the activation of hepatocellular protooncogene triggered OIS and induced CD4^+^ T cells to participate in immune monitoring and immune clearance (13). Therefore, immunosurveillance-dependent senescence-induced therapy is a promising method for the suppression of tumorigenesis.

## Definition of Hepatocellular Senescence

As with other body organs, the liver undergoes a process of aging. Along with aging and upon the conditions of various stressors, such as oxidative stress or oncogene activation, there are many changes in the liver, including decrease in size and in total numbers of normal hepatocytes, decline in regenerative and metabolic capacity, and increase in proportion of polyploid and multinucleated hepatocytes. It is confirmed that the liver of aging mice accumulated polyploid or aneuploid senescent hepatocytes, which is associated with accumulation of DNA damage and activation of INK4a/ARF locus (10, 39).

Hepatocellular senescence inhibits the proliferation of damaged hepatocytes, ensures a stable arrest of proliferation and division, and further causes the alterations of microenvironment and homeostasis (40). Of note, except that more proportion of polyploid or aneuploid exists in senescent hepatocytes (10), manifestation of hepatocellular senescence is non-specific compared with other cellular senescence. Some widely used markers or common events for identifying typical hepatocellular senescence include telomere shortening or dysfunction, SA-β-Gal activity, SASP secretion, cell proliferation arrest, cell enlargement, and increased expressions of p21 and p16^INK4a^. In addition, senescence-related heterochromatic foci and histone γ-H2AX foci exist in the nuclei of some senescent cells, which activate proliferative genes and respond to DNA damage stress (10, 39, 41). The authors performed the study on the mechanism of hepatocellular senescence and senescent reversion, finding that the proportion of polyploid hepatocytes increased along with aging, and the above characteristics of senescent hepatocytes following transplantation could be reversed by ploidy conversion (10).

The detailed mechanism and biological function of hepatocellular senescence in chronic liver diseases have not been fully elucidated. Enhanced oxidative stress resulting from imbalanced reactive oxygen species (ROS) is the main cause of DNA damage in senescent hepatocytes and exists in chronic liver diseases of aging individuals (42, 43). DNA damage causes the overexpression of cell cycle inhibitors and further halts the proliferation of damaged cells by inducing senescence. The liver can normally repair and regenerate if the damage is mild. However, hepatocytes with severe DNA damage lose the capacity of regeneration, and necrosis, apoptosis or senescence will occur (5, 22).

Hepatocellular senescence can cause remarkable changes in tissue homeostasis and microenvironment *via* SASP, which may serve as an antitumor role. At the early stage of chronic liver injury, hepatocellular senescence may serve a protective role by blocking the proliferation and promoting DNA repair of injured hepatocytes, which would reduce the risk of these affected cells becoming cancer cells (15), revealing that early induction of hepatocellular senescence is beneficial to the inhibition of hepatocarcinogenesis. With the assistance of SASP, hepatocellular senescence can recruit and activate immune cells. Activated immunocytes help to clear senescent precancerous hepatocytes, namely senescence surveillance, ultimately preventing malignant transformation (13, 14). Companied with additional mutations such as p53 mutation, senescent hepatocytes contribute to invasive HCC (44). Recovery of wildtype p53 in HCC can induce the activation of immune cells and the elimination of senescent hepatoma cells (11, 45). Kang et al. found that CD4^+^ T cells removed senescent premalignant hepatocytes in association with activated monocytes and macrophages (13), which also indicated the importance of immunosurveillance as an anti-HCC barrier in senescence-induced therapy.

## The Potentially Protective Role of Hepatocellular Senescence Against the Occurrence or Development of Hepatocellular Carcinoma

During the life span, senescence is a common biological phenomenon existing in normal somatic cells and tissues. Of note, senescence is also an unneglectable biological event in tumors (46). Senescence-based therapeutic methods can induce premature senility of cancer cells by the activation of senescence signaling pathways and subsequent SASP (11, 14, 47, 48). Previous studies provided sufficient evidence on the induction of senescence in series cancer cell lines by genetic, chemical, radioactive, as well as biological ways, which supports the consideration of senescence induction as an anti-cancer therapy (24, 49). In 5-aza-2-deoxycytidine-treated HCC cell lines, the induction of p16^INK4a^ upregulation, pRB dephosphorylation, and G1 arrest was indicated by positive SA-β-Gal staining (49).

In recent years, more and more attention has been paid to the relationship between hepatocellular senescence and hepatocarcinogenesis. Accumulating evidence has gradually demonstrated that hepatocellular senescence exhibits anti-HCC effect in specific liver microenvironment. In support of this view, one study reported that inhibition of SIRT6 expression could promote the expressions of p21 and p16 through its regulation of ERK pathway, thereby inducing cellular senescence and reducing the tumorigenicity of hepatoma cells (50). In mice with the deficiency of senescence signaling pathways, hepatocytes suffering from liver injury factor CCl_4_ did not appear senescent phenotypes due to the impairment and disorder of hepatocellular senescence, but turned to the characteristics of liver fibrosis and cirrhosis, finally developing into HCC (51). Our cooperative study found that DUSP16 was upregulated in HCC, which could make HCC cells escape from senescence by inhibiting p53/p21-RB and p16^INK4a^-RB pathways, thus facilitating the proliferation of HCC cells (52).

Moreover, Xue et al. claimed that oncogene H-ras was highly expressed but p53 expression was inhibited in murine hepatocarcinomas with excessive proliferation of HCC cells upon transplantation into the livers of athymic mice. However, these tumors rapidly regressed following the recovery of p53 expression (11). These observations may reveal that hepatocellular senescence contributes to inhibiting oncogene-activated liver cancer. On the other hand, HCC cells driven by Myc in Tet-o-MYC mice exhibited senescent phenotypes after MYC was inhibited, and HCC regressed when p53 was expressed again (53). As another example, oncogene c-Myc downregulation and senescent induction as a result of the response to TGF-β occurred in several HCC cell lines (31), implying that senescent induction may also be linked to the inactivation of oncogene.

The progression of liver diseases is closely related to the characteristics of liver injury and the pathological phenotype or fate of hepatocytes (carcinogenesis or senescence) is determined by the degree of hepatocellular damage. The diseased liver suffering chronic and mild damage to hepatocytes can regenerate and remodel repeatedly by itself until its repair potential is exhausted, which develops into liver cancer after a long period of time. However, acute and severe liver injury as a consequence of harmful stress factors will cause senescence in most hepatocytes in a short time, even leading to fulminant liver failure (22, 54).

At present, the aspect of HCC-relevant research combining with the pathological basis of chronic liver injury is still weak due to the limitation of animal model. The authors have already introduced and applied fumarylacetoacetate hydrolase (Fah) knockout (Fah^−/−^) mice (55, 56) as an ideal animal model of inducible liver injury and even HCC (10, 15) since HCC as a result of chronic liver injury in Fah^−/−^ mice is highly overlapped with the genetic characteristics of human HCC (57). In line with the previous results, the authors demonstrated that Fah^−/−^ mice with chronic liver injury were characterized by the inhibition of hepatocellular senescence and high rate of HCC tumorigenesis, while Fah^−/−^ mice under acute liver injury were characterized by accelerated hepatocellular senescence without HCC occurrence (15). Furthermore, hepatocarcinogenesis under chronic liver injury was significantly restricted due to hepatocellular senescence following the reactivation of acute liver injury in Fah^−/−^ mice (15), revealing the potential antitumor effect of inducible senescence in precancerous hepatocytes.

Of note, how immunosurveillance prevents HCC following senescence induction is still under exploration and not fully understood. Overexpression of p53 in p53 deficient HCC could cause the senescence of HCC cells again, which were further cleared by SASP-activated immune surveillance so as to restrain HCC at last (11). In another study, hepatocytes with the overexpression of Ras rapidly underwent protooncogene-induced senescence, and meanwhile secreted SASP factors to activate immune system to remove themselves, thus restricting the occurrence of HCC (13). It was demonstrated that M1 polar macrophages could promote the elimination of tumor cells (58). Besides, some studies have confirmed that senescent astrocytes expressing p53 could release regulatory factors, promote macrophages to the polarization of type M1 with antitumor effect, and contribute to the formation of antitumor microenvironment (59). Similarly, our study also proved that acute injury-reactivated hepatocellular senescence activated immunosurveillance and promoted the activation and recruitment of NK cells and macrophages by activating CD4^+^ Th1 cells, thus eliminating senescent precancerous hepatocytes and further inhibiting hepatocarcinogenesis in Fah^−/−^ mice under chronic liver injury (15), suggesting that tumor suppression to some extent is resulting from intensive induction of senescence, and subsequent immune-mediated clearance of senescent cells. Collectively, the above hepatocellular senescence-induced immune surveillance has gradually become a potentially effective approach for HCC prevention and treatment.

## The Function and Mechanism of Hepatocellular Carcinoma Inhibition by Hepatocellular Senescence-Induced Immune Surveillance

As stated earlier, sufficient evidence reveals that cellular senescence plays a pivotal role in limiting tumorigenesis and development by immunomodulation (60). The occurrence and development of HCC is a complex and irreversible process, which usually goes through various stages of liver injury, liver fibrosis, liver cirrhosis, and liver cancer (61). Early interruption of either liver injury or fibrosis is the most important steps in inhibiting the progression of liver cancer.

Chronic liver injury is the initial pathological change that develops to HCC, which is typically characterized by progressive destruction and regeneration of hepatic tissues (3, 4). Since cellular senescence exhibiting permanent cell cycle arrest is a potential mechanism of tumor inhibition, significant induction of hepatocellular senescence under chronic liver injury through the treatment with DNA damage chemicals or specific cell cycle inhibitors is beneficial for limiting hepatocarcinogenesis (28, 48). In addition to small molecular reagents that induce cellular senescence, some other treatments are gradually being used, including selecting cytokines/chemokines to activate immunosurveillance, supplementing adoptive immunocytes to remove senescent cells, and screening chemical drugs such as senolytics to induce apoptosis of senescent cells or accelerate clearance of senescent cells (28, 37, 48, 62).

One of the characteristics of cellular senescence is the secretion of a variety of factors such as chemokines or cytokines, the main part of secretory components in senescent hepatocytes, which function as the important immunomodulators (63). The protective attribute of SASP is now known as senescence surveillance since it facilitates the selective clearance of precancerous and cancer cells in liver (37). Therefore, senescent cells are now thought to be antitumorigenic because they restrict tumor cell growth by permanently entering cell cycle arrest, and secretory components from senescent hepatocytes can also recruit immune cells to eliminate damaged hepatocytes under chronic liver injury (25). Generally, under non-cell-autonomous modulation of senescent hepatocyte-secreted SASP factors, a variety of immunocytes and subpopulations are recruited and activated to participate in immunosurveillance, and identify and eliminate damaged precancerous hepatocytes, and thereby preventing the formation of HCC. Hence, containing multiple SASP factors and various types of immunocytes, the tumor immune microenvironment plays a fundamental role in the regulation of senescent response in HCC (37). Th1 lymphocytes provoke senescence induction in target cells through IFNγ and TNFα release (64), and M1 polarized macrophages induce a senescence response mediated by TGFβ signaling (65). In contrast, tumor-infiltrating myeloid-derived suppressive cells (MDSCs) hinder senescence induction and spread through the secretion of interleukin 1 (IL-1) receptor antagonist within tumor microenvironment, and thereby interfering with IL-1α signaling pathway (66). Tumor-infiltrating immune subsets, such as NK cells, M1 macrophages, and Th1 cells, contribute to tumor regression by promoting the clearance of senescent premalignant hepatocytes (15). Moreover, adenovirus-delivered oncogene Ras induced hepatocellular senescence and leaded to activating immune surveillance by recruiting various immune cells (such as macrophages, CD4^+^ Th cells, neutrophils, and NK cells) to infiltrate within the sites of damaged hepatocytes, which repressed precancerous lesions of HCC (13).

Another beneficial feature of hepatocellular senescence is its anti-fibrosis effect on the liver. The senescent process of hepatic stellate cells (HSCs) is closely related to the homeostasis of liver tissues and the formation of hepatic fibrosis. HSCs are located in the Disse space, which usually remain quiescent but become activated only when liver injury occurs. Activated HSCs contribute to fibrotic process following liver damage. The formation of fibrosis is divided into the three steps: first, activation and differentiation of HSCs to α-SMA-positive myofibroblasts; second, deposition of extracellular matrix (ECM), including infiltrative secretion of collagen and TIMP to the injured position; at last, activated HSCs undergo cellular senescence and activate immune surveillance to further eliminate senescent HSCs, or directly undergo apoptosis to be cleared (67). The process of cellular senescence often causes the activation of tumor suppressors p53 and p16. In the model of CCl_4_-induced cirrhosis with deficiency of p53 or p16, Lowe et al. reported that activated HSCs could continuously deposit ECM in the absence of cellular senescence, resulting in severe liver fibrosis (51). In addition, it was also found that senescent HSCs were very helpful since they played a powerful immunomodulatory role in recruiting immune cells, such as macrophages, at the location of damaged tissues. Besides, recruited immunocytes remove senescent cells and also contribute to dissolving fibrotic lesions (51, 68, 69). Therefore, cellular senescence exhibits the function of anti-fibrosis that helps to recover injured liver tissues.

## Intervention Strategies in Senescence-Related Therapy of Hepatocellular Carcinoma

**Figure 1:**
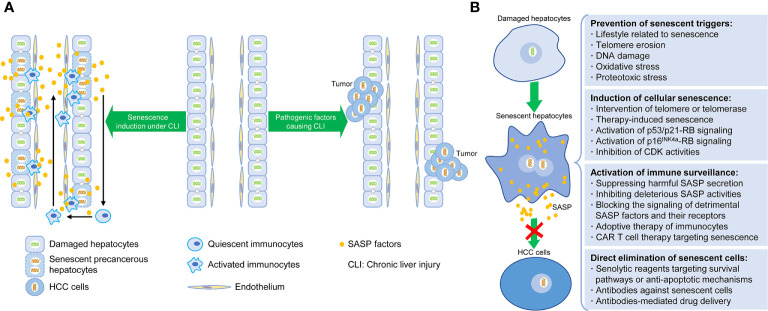
Senescence-induced therapy for HCC. **(A)** Schematic diagram depicting the relationship between hepatocellular senescence and hepatocarcinogenesis under chronic liver injury. **(B)** Intervention strategies targeting hepatocellular senescence to inhibit hepatocarcinogenesis and extend healthy life span, including preventative steps for senescent triggers, induction and clearance of senescent hepatocytes, and activation of senescence-related immunosurveillance.

In accordance with the existing researches, the induction of senescent cells, the regulation of SASP, and the clearance of senescent cells are the three major senescence-targeted strategies of HCC intervention (37, 62, 63, 70, 71). The corresponding contents are described below and summarized in **Figure 1**.

### Induction of Senescent Hepatocytes by Small Molecules

As mentioned earlier, therapy-induced senescence (TIS) has become a potential antitumor scheme. Targeted therapies for HCC can be explored from the perspective of senescent induction. There are mainly two ways to induce cellular senescence: one is to induce cell replicative senescence through the intervention of telomere or telomerase; the other is to induce premature senescence through specific factors activating p53/p21-RB or p16^INK4a^-RB signaling pathways, finally limiting the entry into cell cycle and blocking the ability of division and proliferation (17).

Even though the senescent signals of p53/p21 and p16^INK4a^ were significantly activated following the induction of acute liver injury, which repressed chronic injury-induced hepatocarcinogenesis, it was found that acute liver injury meanwhile caused an increasing mortality of Fah^−/−^ mice and poor controllability of treatment, which makes the difficulty of clinical translation. At present, development, screening, and application of specific small molecular compounds to induce cell cycle arrest for HCC prevention and therapy has been a promising field (28). For instance, lysine acetyltransferases (KATs)-catalyzed histone acetylation plays an essential role in chromatin organization and function. Chromosomal translocations of oncogenes KAT6A/B encoding for KATs were identified in a variety of cancers and KAT6A accounted for senescent suppression by regulating the suppressors of CDKN2A locus. Therefore, histone acetylation inhibitors WM-8014/1119, targeting KAT6A/B, could effectively inhibit the growth of tumor cells through inducing their senescence without causing DNA damage (72). Recent study by Wang et al. showed that cell division cycle 7-related protein kinase (CDC7) inhibitor XL413 specifically induced the senescence of HCC cells with p53 gene mutation, but exhibited no effect of senescent induction on normal cells, and therefore it could specifically eliminate HCC cells (73).

Of note, since CDKs as downstream targets of p53/p21 and p16 signals can trigger cell cycle from G1 phase to S phase, direct inhibition of CDK activities can also induce cells to deviate from normal cell cycle and become senescent (28). Small molecule compound Palbociclib can specifically block the phosphorylation of RB protein initiated by CDK4/6, upon binding to the active site of CDK4/6, which causes cell cycle arrest in G1 phase (74). Palbociclib can induce the senescence of both tumor and normal cells *in vitro* and *in vivo* (75, 76). A study of Palbociclib treatment on a variety of human HCC cell lines suggested that cellular senescence occurred in HCC cell lines with normal RB protein function (77). Besides, the authors utilized Palbociclib as a senescent inducer to stably cause hepatocellular senescence and effectively inhibit hepatocarcinogenesis without affecting liver function in Fah^−/−^ mice with chronic liver injury, showing its biological safety and feasibility in clinical application (unpublished data). Cell cycle inhibitors for potential prosenescence therapies are listed in **Table 2**.

**Table 2:** Candidate reagents for senescent cell induction.

Small molecule	Targeted protein	Reference
CVT-313, CVT-2584	CDK2	(78)
Palbociclib	CDK4 and CDK6	(79)
Ribociclib	CDK4 and CDK6	(79)
Abemaciclib	CDK4 and CDK6	(79)
Milciclib	CDKs	(6)
GRN163L	telomerase activity	(80)
Nutlin, RITA	p53-degrading ubiquitin ligase MDM2	(81)
PRIMA-1, MIRA-1	mutant p53 reactivation	(81)
WM-8014, WM-1119	histone acetyltransferases KAT6A/B	(72)
XL413	DNA-replication kinase CDC7	(73)

CDK cyclin-dependent kinase; MDM2, murine double minute 2; KAT6A/B, lysine acetyltransferase 6A/B; CDC7, cell division cycle 7-related protein kinase.

### Activation of Immunosurveillance by SASP Factors or Adoptive Immunocytes

The key point of senescence-induced therapy for HCC is that senescent hepatocytes-produced proinflammatory SASP factors recruit a variety of immunocytes to participate in immunosurveillance to further identify and eliminate senescent cells, and finally inhibit HCC (27, 48). Indeed, CD4^+^ T cells, in the form of T helper cells, could function through monocyte/macrophage system to remove senescent cells and suppress tumor formation (82). Besides, the immune system can initiate an effective antitumor response during the development of HCC, since the progression of chemical carcinogen diethylnitrosamine (DEN)-induced liver tumor was significantly enhanced in T-cell and B-cell immune deficient mice (Rag1^−/−^ mice) (83). Senescence-associated immune responses require the recruitment and maturation of CCR2^+^ myeloid cells, and CCR2 ablation caused outgrowth of HCC (14). Cytotoxic T cells with chimeric antigen receptors (CAR T cells)-mediated therapy has been reported to have therapeutic potential for senescence-associated pathologies. CART T cells targeting uPAR, identified as a specific marker on senescent cell membrane, could effectively ablate senescent cells *in vitro* and *in vivo*. Of note, the therapeutic effect of uPAR-specific CAR T cells on liver fibrosis in mice with nonalcoholic fatty liver disease (NAFLD) was remarkable due to the elimination of senescent hepatocytes (84). Moreover, in Fah^−/−^ mice under chronic liver injury, senescent reactivation of precancerous hepatocytes could recruit immunocytes such as M1 type macrophages, CD4^+^ Th1 lymphocytes, and NK cells and secrete SASP factor CCL2, which played an inhibitive role against tumorigenesis (15). **Table 3** showed the types of immunocytes involved in the clearance of senescent cells in different mouse models of liver diseases.

**Table 3:** Candidate reagents for SASP modulation and potential immunocytes for immunosurveillance.

Small molecule	Targeted SASP pathway	Reference
Metformin	NF-кB	(85)
UR-13756, BIRB 796	p38 MAPK/MK2	(86)
Simvastatin	Rho family GTPases	(87)
Sertraline, Rapamycin	mTOR	(73, 88)
**Antibody**	**Targeted SASP ligand/receptor**	**Reference**
Adalimumab/Infliximab	TNFα	(89)
Etanercept	TNFα	(90)
Canakinumab	IL-1β	(91)
Rilonacept	IL-1α and IL-1β	(92)
Anakinra	IL-1R	(93)
Siltuximab	IL-6	(94)
Tocilizumab	IL-6R	(95)
**Immunocyte**	**Animal model**	**Reference**
monocyte-derived macrophages and CD4^+^ Th1 cells	Nras^G12V^-transfected mouse model of HCC	(13)
CD4^+^ T cells, monocytes, and macrophages	liver-specific MYC oncogene transgenic mouse model of HCC	(82)
NK cells	p53^−/−^; INK4a^−/−^ARF^−/−^ mouse model of CCl_4_-induced hepatic fibrosis	(51)
neutrophil cells, NK cells, and macrophages	p53^−/−^ mouse model of HCC	(11)
CD4^+^ Th1 cells, NK cells, and macrophages	Fah^−/−^ mouse model of HCC under chronic liver injury	(15)
Senolytic CAR T cells	mouse model of CCl_4_ or NASH-induced hepatic fibrosis	(84)

NF-кB nuclear factor kappa B; MAPK, mitogen-activated protein kinase; MK2, MAPKAP kinase-2; mTOR, mechanistic target of rapamycin kinase; TNFα, tumor necrosis factor α; IL-1/6, interleukin-1/6; IL−1/6R, interleukin−1/6 receptor; HCC, hepatocellular carcinoma; Th1 cells, T helper 1-type cells; NK cells, natural killer cells; CAR T cells, chimeric antigen receptor T cells; INK4a, p16 or cyclin-dependent kinase inhibitor 2A; ARF, ADP ribosylation factor; Fah, fumarylacetoacetate hydrolase; CCl_4_, carbon tetrachloride; NASH, non-alcoholic steatohepatitis.

Of note, it was found that CD8^+^ T cells-mediated adaptive immune system played a crucial role in promoting malignant transformation of hepatocytes and the formation of HCC through lymphotoxin β signal in Fah^−/−^ mice (57). Besides, SASP cytokines IL-6 and IL-8 and their signals exhibited detrimental paracrine effects of prolonged presence of senescent cells and caused tumor initiation, progression, and metastasis (63, 96). These researches demonstrate that the differences of immune microenvironment under specific conditions may exhibit the similar or opposite effect on HCC progression and emphasize that senescence-related immune surveillance should be strictly tuned to balance immune surveillance and cancer risk or amplify the net antitumor effect. For example, the development of deleterious SASP-neutralizing approaches can be considered from the following aspects: inhibiting pro-SASP signaling pathways within senescent cells, blocking the secretion of harmful SASP factors, and suppressing the activities of specific SASP factors or their receptors (**Table 3**).

### Elimination of Senescent Hepatocytes by Senolytic Drugs

In addition to the above-mentioned immune surveillance-based strategies, senescent cell elimination is another way of senescence-targeted HCC therapy. In view of the fact that the secretory phenotypes of inducible senescent cells may bring about potential adverse effects in the process of HCC development, such as persistent senescence-caused formation of tumor microenvironment, the removal of existing senescent cells by direct killing through pharmacological intervention, namely senotherapies, is a straightforward means of anti-HCC (18, 97). As discovered by researchers from Mayo Clinic, the advantages of currently screened senolytic drugs, such as ABT-263 (Navitoclax) (98), Dasatinib (99), Quercetin (99), DRI-FOXO4 (100), UBX0101 (101), and AP20187 (102), are that they can selectively target senescent cell anti-apoptotic pathways (SCAPs), accelerate senescent cell apoptosis, and specifically eliminate senescent cells. Senescent cells often release a series of anti-apoptotic signals to promote survival, such as ephrins (EFNB1 and EFNB3)/dependence receptors, PI3K/AKT, Bcl-2 (Bcl-xl, Bcl-2, and Bcl-w), p53/FOXO4/p21/Serpins (PAI-1 and PAI-2), HIF-1α, and HSP90 (23, 62). These signal factors can enhance the ability of anti-apoptosis in senescent cells and further lead to local inflammation, and even cause dysfunction of normal tissue function. ABT-263 is a pan-inhibitor of Bcl-2 family, which can selectively eliminate senescent cells with high expressions of Bcl-2, Bcl-XL, and Bcl-W. Dasatinib (D), a small molecule broad-spectrum inhibitor of Src family protein tyrosine kinases, can induce the senescence of epithelial and adipocytes. Quercetin (Q), belonging to bioflavonoids and antioxidants, can target Bcl-2, HIF-1 α, PI3K, and p21. The combination of D and Q (D + Q) has the potential to act on multiple SCAP targets and selectively promote the clearance of senescent cells by accelerating apoptosis in multiple tissues (99, 103). The regimen of ABT-263 or D + Q suppressed tumor progression by eliminating senescent HSCs that promoted tumor growth in the liver of hepatocyte-specific FBP1^−/−^ mice (104). The small molecule AP20187 could induce apoptosis through FKBP-Casp8 fusion protein dimerization in an aging-related model of INK-ATTAC mice (105), which leaded to the elimination of p16^INK4a^-positive cells. It was also used to clear senescent cells to reverse age-dependent hepatic steatosis. This method was equally successful, compared to D + Q therapy, in inhibiting senescence and reducing liver fat accumulation induced by high-fat diet in mice (102). Currently, as searched in the ClinicalTrials.gov database, senolytic drugs are being tested in human clinical trials for the treatment of osteoarthritis (Identifier: NCT04210986), idiopathic pulmonary fibrosis (Identifier: NCT02874989), and chronic kidney disease (Identifier: NCT02848131) (99, 106, 107). However, senolytic drug therapy can cause extra-target effects in addition to exhibiting targeting functions of senescent cell clearance. For example, senolytic drugs targeting Bcl-2 family members have toxic effects on some immunocytes and platelets, and may cause thrombocytopenia and lymphopenia (97). Therefore, increasing the selectivity of these compounds by targeting more specific mechanisms of senescence may reduce the toxicity.

Except for senolytics, Cai et al. reported β-galactosidase-targeted prodrug SSK1 as a new anti-senescence compound. SSK1 has no toxic effect, but it can be metabolized and activated to be toxic molecule by activity-enhanced SA-β-Gal in senescent cells, subsequently inducing senescent cell death and reversing hepatic fibrosis and other senescent phenotypes of aging mice with no effect on normal cells (108). In the study by Wang et al., sertraline, a drug used in clinical treatment of depression, could specifically promote the apoptosis of CDC7 inhibitor-induced senescent hepatoma cells through downregulating mechanistic target of rapamycin (mTOR) signaling pathway (73). Results from animal models and clinical samples of HCC demonstrated that the combination of CDC7 inhibitor and mTOR inhibitor could significantly inhibit the progression of HCC, and its antitumor effect was significantly better than that of non-specific multi-target drug Sorafenib (73). In terms of antitumor effect, the model of first induction and subsequent elimination of senescent tumor cells maybe more effective than single one of the two anti-HCC methods. The small molecules that have been reported for the use of senescent cell clearance are listed in **Table 4**. In summary, promoting the clearance of senescent hepatocytes may have the potential to become an innovative approach for the prevention or treatment of HCC since there are few reports on this aspect, especially in HCC under chronic liver injury.

**Table 4:** Candidate reagents for senescent cell clearance.

Small molecule	Targeted pro-survival protein/pathway	Reference
ABT-737	Bcl-XL, Bcl-W	(109)
ABT-263 (Navitoclax)	Bcl-2, Bcl-XL, and Bcl-W	(98, 110)
A1331852, A1155463	Bcl-XL	(111)
Dasatinib	RTKs	(99)
Quercetin	PI3K/Akt, Bcl-2, HIF-1α, and p21	(99)
DRI-FOXO4	disruption of p53/FOXO4 interaction	(100)
UBX0101	MDM2	(101)
AP20187	dimerization of FKBP-fused Casp8	(102)
SSK1	SA-β-Gal and p38 MAPK	(108)
17-DMAG (Alvespimycin)	HSP90	(112)
Fisetin	PI3K/Akt	(111)
Phloretin	glucose and fatty acid metabolism	(113)
Panobinostat	HDACs	(114)
Cytochalasin B	AMPK and autophagy	(113)
Etomoxir	AMPK and autophagy	(113)
Sodium oxamate	AMPK and autophagy	(113)

Bcl-2 B cell lymphoma 2; Bcl-XL, B cell lymphoma XL; Bcl-W, B cell lymphoma W; RTKs, tyrosine kinase receptors; PI3K, phosphatidylinositol-4,5-bisphosphate 3-kinase; Akt, PKB or protein kinase B; HIF-1α, hypoxia inducible factor-1α; DRI-FOXO4, d-retro-inverso peptide-forkhead box protein O4; MDM2, murine double minute 2; FKBP, FK506-binding protein; Casp8, Caspase 8; SSK1, senescence specific killing compound 1; SA-β-Gal, senescence-associated β-galactosidase; MAPK, mitogen-activated protein kinase; HSP90, heat shock protein 90; HDACs, histone deacetylase inhibitors; AMPK, adenosine monophosphate (AMP),-activated protein kinase.

## Dual Effects of Hepatocellular Senescence on the Occurrence and Development of Hepatocellular Carcinoma

There are two opposite views explaining the biological effect of cellular senescence. Cellular senescence is considered as a natural mechanism of anti-cancer since senescence can cause arrest of cell division and proliferation, indicating that senescence may be a beneficial event in the body (27, 48). On the other side, cellular senescence may lead to the decline of regenerative ability of tissues or organs (62). Thus, it may impede functional and organizational renewal and thus becomes a deleterious process in the body. Along with the increase of age, accumulative senescent cells may lead to aging process, organ dysfunction, and even aging-related diseases such as cancer, stroke, atherosclerosis, type 2 diabetes, Alzheimer’s disease, cataract, and osteoporosis (18, 62). In the future, how to balance the two aspects of cellular senescence so that we can better utilize its antitumor effect in the prevention and treatment of HCC while avoid its detrimental aspect, is worth to be studied in depth.

Recent literature reported that the degeneration of immune surveillance and clearance system in the elderly is one of the important causes of cancer formation (115). Along with aging, not only the accumulation of mutated cells undergoing irreversible injury, but also the metabolic abnormalities and functional decline of immune system in the organism will increase the risk of cancer (62). Therefore, aging-caused weakening of immune surveillance may be another important factor for tumorigenesis. Indeed, compared with young mice, chronic and slight liver injury in one-year-old Fah^−/−^ mice will spontaneously develop HCC (116), which warrants further investigation of the changes in the type, proportion, and metabolism of immunocytes such as CD4^+^ Th cells, macrophages, and NK cells.

The correlation between senescent cells and immune system is mediated by SASP. Although SASP-regulated immune response plays a crucial role in the prevention and treatment of HCC, it is quite a complex process since SASP factors have highly dynamic changes in expression and composition over the period of senescence, which depends on the mode of senescence induction, the cell type, the duration of senescence, and active signaling cascades. In different situations, immune signaling system following the induction of hepatocellular senescence exhibits the distinct effects on the progression of HCC (117, 118). As mentioned above, under early chronic liver injury, hepatocellular senescence plays a protective role against hepatocarcinogenesis *via* immunosurveillance mechanism. However, in the late stage of chronic liver injury, single and scattered HCC nodules appear. Existing senescent cells secrete SASP factors including proinflammatory cytokine/chemokines such as IL-1, IL-6, and IL-8 at an abnormal level, which can promote a large number of transformations from non-senescent cells to senescent cells in a paracrine manner (117). It is possible that chronic liver injury causes progressive and repetitive liver destruction and regeneration, which leads to the accumulation of shorter telomeres in hepatocytes and further results in accumulative hepatocellular senescence (119). In this case, the microenvironment created by SASP secretory factors is more suitable for the survival of hepatoma cells, eventually promoting the formation and development of HCC (117).

Recently, using Fah^−/−^ mouse model of HCC, the authors demonstrated that Palbociclib-induced hepatocellular senescence effectively restricted the occurrence of HCC following Palbociclib administration in the early-phase, but surprisingly enhanced the development of HCC in the late period of drug treatment. The next findings revealed that hepatocellular senescence at early precancerous stage could eliminate atypical hyperplastic hepatocytes and restrain hepatocarcinogenesis through SASP-activated immunosurveillance. However, in HCC development period, compared with early stage, the emergence of much more senescent hepatocytes may secrete a large amount of SASP factors, which caused the changes in tumor microenvironment and accelerated HCC progression (unpublished data).

Hence, induction of tumor cellular senescence is a double-edged sword, which may not only inhibit the occurrence of tumor, but also accelerate the development of tumor, especially in aging body (16, 120–122). Administration of small molecule compounds targeting cellular senescence induction or SASP modulation could become rather complicated. In the future, the investigation of mechanism on cellular senescence regulating the occurrence and development of HCC, the exploration of scheme on inducing hepatocellular senescence, and the construction of favorable antitumor microenvironment will become new research hotspots. In addition, it is also important that more attention should be paid to the choice of optimum medication time and clinical safety assessment of small molecule compounds inducing or inhibiting hepatocellular senescence.

## Conclusion and Future Prospects

In recent years, cellular senescence has aroused great interest in the research of HCC. It can be mediated by various pathways and molecules, just like cell death. Cell cycle suppressor-induced senescence may possess the antitumor mechanism on atypical hyperplastic hepatocytes, which highlights its clinical significance. On the other hand, inducible or spontaneous senescence observed in HCC will help to explore new methods of HCC prevention and treatment. The following is the summary points:

1. Senescence is characterized by a number of phenotypes and closely involved in the pathogenesis of age-related diseases including HCC (**Table 1**). Established senescence markers such as p16^INK4a^, p21, and SA-β-Gal can be used to identify senescent cells, but these markers are representative rather than specific. Hopefully, more reliable biomarkers could be investigated to selectively identify and target senescent cell subtypes with harmful secretory phenotypes while maintaining other subtypes with beneficial secretory phenotypes for the suppression of tumor occurrence and development. Meanwhile, screening and examination of circulating SASP factors may be helpful to evaluate the efficacy of senescence-targeting therapy.
2. SASP entails continuous secretion of various proinflammatory factors and have highly dynamic changes in expression and composition over the senescent process. Heterogeneity of SASP can cause both beneficial and deleterious effects on age-related diseases including HCC, each of which depends on the physiological and pathological context in different periods of diseased organs including liver.
3. Hepatocellular senescence is regarded as a stress response that inhibits liver tumorigenesis early in lifespan, but it may become a basic harmful process along with the age growth that drives the accumulation of persistent age-relevant pathologies (e.g., local or systemic inflammation, impaired regenerative capacity, and weakened immune surveillance) and subsequent emergence of hepatoma cells failing to enter cell cycle arrest late in lifespan, and even fuels advanced and recurrent liver cancer. Accordingly, it can also be considered that transient presence of senescent hepatocytes may be beneficial while their chronic presence may be detrimental.
4. The positive functions of hepatocellular senescence and accompanying SASP contribute to proposing new clinical strategies of HCC-targeted therapy, namely senescence-induced therapy in HCC, for the purpose of reinforcing tumor suppressive growth arrest, stimulating immune clearance of senescent hepatocytes, and optimizing the repair of injured liver tissues through specific interventions in several checkpoints of senescence-mediated therapy.

However, there are still issues to be solved in the knowledge of complex roles of cellular senescence and senescence-related anti-HCC therapies in both the occurrence and development of HCC. Future problems are listed below:

1. More intensive search and screening for small molecular compounds that can selectively target the induction or removal of senescent cells with harmful secretory phenotypes, or selectively modulate the SASP. Of note, these reagents inhibiting purely deleterious senescent cells or SASP factors would avoid to disrupt beneficial functions of senescence and proinflammatory processes if they exist and can be screened.
2. More comprehensive understanding of the reason why senescent cells increase along with age and play the role of promoting tumorigenesis during the process of tumor late in lifespan, despite the ability to eliminate themselves through immune system.
3. More comprehensive understanding regarding the optimal situation where senescent cells are beneficial and participate in tumor suppression, tissue repair, and regeneration. Also, cellular senescence induction should be strictly tuned to amplify the net antitumor effect of senescence.
4. More exploration of combining other pharmacological treatment strategies and senescence-induced therapies to enhance the antitumor effect of hepatocellular senescence in HCC, such as the combination of immune-checkpoint inhibitors and CDK4/6 inhibitors, or senescence-inducing drugs and proapoptotic senolytic reagents.

Despite the close relationship of cellular senescence abnormality and HCC pathogenesis, the regulatory roles of senescence pathways in HCC have not been full clarified. It is anticipated that senescence study will attract more attention in the future and provide more promising methods and experience for the aim of translating these senescence-associated therapies to clinical applications. Further elucidation on the molecular mechanisms of cellular senescence and senescence-associated immunotherapy will enable us to make therapeutic options more accurately in the prevention and treatment of HCC.

## Author Contributions

PL wrote the original version of the manuscript. QT contributed to the modification of the main sections. MC provided the suggestions for the content on hepatocellular senescence and senescence-induced therapy. WC contributed to the editing of the section on SASP and senescence-related immunosurveillance. YL offered the assistance for the section on the induction and clearance of senescent cells. ZL reviewed the manuscript and contributed to valuable academic advice. ZH provided the guidance and supervision for conceptual proposal and design, and contributed heavily to the writing and revision of the manuscript in its present form. All authors contributed to the article and approved the submitted version.

## Funding

This work was supported by the Major Program of Development Fund for National Key Research and Development Project (2019YFA0801502), Major Program of Stem Cell and Translational Research for National Key Research and Development Project (2020YFA0112600), Shanghai Zhangjiang National Innovation Demonstration Zone (ZJ2018-ZD-004), National Natural Science Foundation of China (81772954), the Top-Level Clinical Discipline Project of Shanghai Pudong (PWYgf2018-04), Program of Shanghai Academic/Technology Research Leader (20XD1434000), and Peak Disciplines (Type IV) of Institutions of Higher Learning in Shanghai.

## Conflict of Interest

The authors declare that the research was conducted in the absence of any commercial or financial relationships that could be construed as a potential conflict of interest.
